# Sexual and reproductive health of adolescents in schools for people with disabilities

**DOI:** 10.11604/pamj.2019.33.299.18546

**Published:** 2019-08-14

**Authors:** Mercy Obasi, Stephen Manortey, Kofi Adesi Kyei, Michael Kwabeng Addo, Sharon Talboys, Lynette Gay, Frank Baiden

**Affiliations:** 1 Department of Public Health, University of Utah, West Africa Campus, Kpong, Ghana; 2 Department of Radiography, School of Biomedical & Allied Health Sciences, Accra, Ghana; 3 National Centre for Radiotherapy and Nuclear Medicine, Korle-Bu Teaching Hospital, Accra, Ghana

**Keywords:** Adolescent, assessment, disability, sexual, reproductive health

## Abstract

**Introduction:**

Persons with disabilities have the same sexual and reproductive health (SRH) needs as the abled people but they often face barriers to SRH information and services which are necessary for healthy and safe relationships, protection from HIV and other sexually transmitted infections (STIs). This study sought to access the SRH services among adolescents with disabilities in four Special Needs Schools in Ghana.

**Methods:**

The study adopted a cross-sectional study design with a quantitative approach to data collection between the months of January to March, 2018. A structured and pretested questionnaire was used to collect data from adolescents with disabilities from selected schools in Ghana. Both descriptive and inferential statistics were performed using chi-square test and multivariate logistic regression.

**Results:**

Majority of participants had hearing disability (52.1%). The average age at menarche among females was 13 years whiles the age at which puberty was attained among boys was 14 years. School teachers were the major source of information on SRH for the respondents (63.7%) followed by parents (12.2%). A majority (67.1%) of respondents had good knowledge of SRH. Factors which were significantly associated with knowledge level were age (p=0.026), religion (p=0.034), sources of information (p<0.001), guardians (p=0.049).

**Conclusion:**

The majority of participants had good knowledge of SRH, although their knowledge of contraceptive and access were poor. Only condoms were mostly known. There is the need for increased awareness on the availability of other contraceptives methods and the removal of barriers to contraceptive methods.

## Introduction

Sexual Reproductive Health (SRH) is a state of complete physical, emotional, mental and social well-being in relation to sexuality, in all matters relating to reproductive system and its functions and processes [1]. SRH concerns include maternal and newborn health, family planning, adolescents and youth reproductive health. Other gender related SRH concerns are sexual gender-based violence, HIV and AIDS, sexually transmitted infections (STIs), reproductive tract infections, fertility and cancers of reproductive organs such as breast, cervical, uterine and ovarian cancers. The World Health Organization (WHO) estimates that 15 percent of the global population is persons with disabilities [2]. Globally, almost 180 million young people between the ages of 10-24 live with a physical, sensory, intellectual or mental health disability significant enough to make a difference in their daily lives [2]. A majority of these young people, (80%) live in low-income countries [3, 4]. Other vulnerable groups among people with disabilities are women, children, elderly, refugees, displaced persons and migrant workers [5]. These group of people are usually excluded from most educational, economic, social and cultural opportunities, and often face more discrimination, isolation and abuse than their non-disabled peers (Groce, 2004) [3]. There is growing body of awareness that recognizes that persons with disabilities have historically been denied their sexual and reproductive health rights [6]. Some of these include less access to SRH information, which is necessary for healthy and safe relationships, protection from HIV and other STIs, and realization of sovereignty in family planning decisions [7]. Findings further emphasize the multiple and interrelated forms of discrimination that women with disabilities often experience, many of which increase their vulnerability to different forms of violence [1, 5]. Lack of confidentiality and anonymity, and distance have been identified as key barriers to accessing SRH services, as well as providers' attitudes and communication [8]. These barriers contribute to the disadvantages experienced such as poor health outcome, lower educational achievements, a higher rate of poverty and inability to participate fully in community activities. Sexual relationships are naturally difficult to manage and having a disability makes it more difficult. In most places, society incorrectly believes that youth with disabilities are asexual and cannot be abused [5, 9]. A study conducted by Karimu [10] among visually impaired women in Ghana found that, visually impaired teenagers often engage in pre-marital sex with no adequate knowledge to prevent unplanned pregnancies or sexually transmitted infections and thus results in unwanted pregnancies and dropping out of school. However, few programs are currently available to address the SRH needs of persons with disabilities. Understanding their unique needs, risks and capacities can better ensure that the appropriate authority addresses their SRH rights. This study sought to access the SRH services among adolescents with disabilities in four Special Needs Schools in Ghana. In doing that, an assessment of the knowledge, behavior and practices regarding sexual and reproductive health was conducted. This was done because in low- income countries such as Ghana, participants in these categories are limited with regards to education and access to services, which have been found to be responsible for the inappropriate risk perception and negative social beliefs about reproductive health related issues.

## Methods

A cross-sectional study design with a quantitative approach was adopted to collect data between the months of January to March, 2018. The study focused on the knowledge and practice of SRH among adolescents with disabilities in selected special needs schools in Ghana. A probability sampling technique was used to obtain the respondents for the study. Four schools of people with special needs in two separate regions in Ghana were visited and sampled study participants were interviewed after obtaining administrative approval from the heads of the schools and informed consent from qualified participants. The study population involved adolescents aged 15-19 years who were enrolled in the purposively selected schools. Students with visual and hearing impairment and intellectual disabilities were randomly selected for the study. The target sample size was determined based on the following assumptions; an expectation of the prevalence of sexual activities among the respondents to be 50% (selected to achieve maximum variance) and the desire to estimate the prevalence among the group with a 5% margin of error at a significance level of 95% (α=0.05) assuming that adolescents with disabilities were selected among the population of 5,000, thus the preferred sample size was 357. In order to determine the required sample size from each of the four schools, the population proportion of each school was determined. A systematic random sampling technique using the schools' students' register was employed to achieve the required sample size for the study participants. Three categories of disabilities such as visual, hearing, and intellectual were included in the study. A face-to-face pretested structured questionnaire adapted from WHO Adolescent sexual and reproductive health survey, [4] and Ghana Adolescent survey, [11] was designed for the study. The questionnaire contained only close-ended questions. The data was collected using an electronic data collection device by the research participants. The questionnaire covered socio-demographic information as well as information on SRH knowledge, access to contraceptives, relationship and sexual behavior. Data collected was coded using Ona collect and managed in electronic data collection database. The coded data was imported into STATA statistical software package [12] for analysis. Uninvariate analyses were performed to offer a general descriptive overview of the socio-demographical variables in the dataset. A Pearson's Chi-square test and logistic regression was used to assess the associations between the dependent and independent variables. A p-value <0.05 was considered as statistically significant at 95% confidence level. Ethical approval was sought from the Ethics and Protocol Review Committee of a higher institution and the head of unit of the study site before data collection. Written informed consent was obtained from the Special Schools as well as teachers before data collection. A detailed explanation of the procedure and its purpose were made known to the eligible participants to obtain their consent. The questionnaires were coded and thus, devoid of participants' identification particulars to ensure anonymity. Only pseudonymized data was analyzed.

## Results

Out of the total of 357 sampled respondents, only 280 of them could appropriately answer the various questions, resulting in a 78% response rate. The analysis was based on a sample population of adolescents with disabilities. The average age of the respondents was 17 years (± 1.36) with the age ranging from a minimum of 15 years to a maximum age of 19 years. The male to female ratio were 50.4% and 49.6% respectively. Students with hearing impairment formed the majority (52.1%), followed by those with visual impairment formed (30.0%). Concerning the sexual and reproductive health information about respondents, the average age at menarche among the female respondents was 13 years (+1.6). Menarche began as early as 9 years and as late as 19 years, among the female respondents. In Table 1, most of the respondents (87.9%) stay with their parents when not in school and 71.1% of them have their parents staying together. Most important sources indicated that school teachers were their major sources of information (63.9%). Other sources mentioned were parents, siblings, and friends which formed 12.2%, 2.5%, and 10.4% respectively (Table 2). Disability type of respondents (p-value 0.057) and the age of respondent (p-value 0.026) were found to be fairly significant (Table 3). Other factors such as sex of the respondent, religion, and education level had no statistical significance with knowledge level. The results showed that age of the respondents, sources of information, religion and whom they stay with were statistically significant. Participants with visual impairment were also found to be 2.3 times more likely to have high knowledge compared to those with intellectual disability holding all other variable constant (p-value, 0.043; AOR: 2.37; 95% C. I: 1.03-5.49) (Table 4).

**Table 1:** Relationship of respondents with guardians

Variables	Categories	Frequencies n (%)
**Stay with Guardian**		
	Parent	246 (87.9)
	Other relatives	14 (5.0)
	Non-relatives	20 (7.1)
**Parents living together (n=246)**		
	Yes	175 (71.1)
	No	71 (28.9)
**Either parent alive**		
	Yes	256 (91.4)
	No	24 (8.6)
**Communication with Guardians**		
	Very easy	70 (25.0)
	Easy	99 (35.4)
	Average difficult	32 (11.4)
	Very difficult	70 (25.0)
	Do not see him/her	9 (3.2)
**Daily activities when not in school**		
	Studying/homework	63 (22.5)
	Household chores	90 (32.1)
	Helping with family business	23 (8.2)
	Work to get money	17 (6.1)
	Playing/Chatting with friends	51 (18.2)
	Idling	26 (9.3)
	Others	10 (3.6)

Data is presented in frequencies and percentages. Sources: Field data, 2018

**Table 2:** Sources of information on SRH among adolescents with disability (AWD)

Order of Importance	Sources of information on SRH	Frequencies n (%)
**First most important source of information**		
	School teachers	179 (63.9)
	Parents	34 (12.2)
	Siblings/Other family members	7 (2.5)
	Friends	29 (10.4)
	Health personnel	1 (0.4)
	Media	15 (5.4)
	Others	15 (5.4)
**Second most important source of information**		
	School teachers	97 (34.6)
	Parents	50 (17.9)
	Siblings/Other family members	20 (7.1)
	Friends	56 (20.0)
	Health personnel	6 (2.1)
	Media	29 (10.4)
	Others	22 (7.9)

Data is presented in frequencies and percentages. Sources: Field data, 2018

**Table 3:** bivariate analysis of socio-demographic factors associated with knowledge among respondents

Variables	Knowledge Scores		P-Value
	Good n (%)	Lown (%)
**Age of respondents**			
15-17	92 (61.3)	58 (38.7)	0.026*
18-19	96 (73.9)	34 (26.2)	
**Sex**			
Male	98 (69.5)	43 (30.5)	0.397
Female	90 (64.8)	49 (35.3)	
**Disability type**			
Intellectual	31 (62.0)	19 (38.0)	
Hearing Impairment	92 (63.0)	54 (37.0)	0.057
Visual Impairment	65 (77.4)	19 (22.6)	
**Educational Level**			
Basic	140 (68.0)	66(32.1)	
Secondary	38 (70.4)	16 (29.6)	0.225
Rehabilitation	10 (50.0)	10 (50.0)	
**Religion**			
Christian	173 (69.2)	77 (30.8)	0.034 *
Muslim	15 (50.0)	15 (50.0)	

Test of association using chi-square

**Table 4:** multivariate analysis of factors associated with knowledge among respondents

Variables	Categories	P-Value	OR (95%)	P-Value	AOR (95%)
**Age of respondent**					
	15- 17 years 18-19 years	Ref 0.0265*	1 1.78 (1.06-4.86)	Ref 0.028*	1 1.88 (1.07-3.30
**Disability type**					
	Intellectual	Ref	1	Ref	1
	Hearing	0.8984	1.04 (0.54-2.03)	0.226	1.61 (0.75-3.46)
	Visual	0.0570	2.10 (0.96-4.57)	0.043*	2.37 (1.03-5.49)
**Sources of information on SRS**					
	Friends	Ref	1	Ref	1
	School teacher	< 0.0001*	4.62 (1.98-10.80)	<0.001*	4.46 (1.84-10.82)
	Relatives	0.2284	1.81 (0.68-4.82)	0.094	2.39 (0.86-6.66)
	Others	0.4311	1.51 (0.54-4.425)	0.374	1.64 (0.55-4.85)
**Religion**					
	Muslim	Ref	1	Ref	1
	Christian	0.0347*	2.25 (1.04-4.86)	0.037*	2.45 (1.05-5.67)
**Guardian**					
	Parents	Ref	1	Ref	1
	Other relatives	0.6257	1.34 (0.42-4.42)	0.632	1.36 (0.38-4.84)
	Non-relatives	0.0228*	4.84 (1.08-21.72)	0.040*	5.15 (1.08-24.58)

Ref: reference group; OR: Crude odds ratio; AOR: Adjusted odds ratio; statistical significance is measured at p-value<0.05 and

* : the measurement is statistically significant

**Figure 1:**
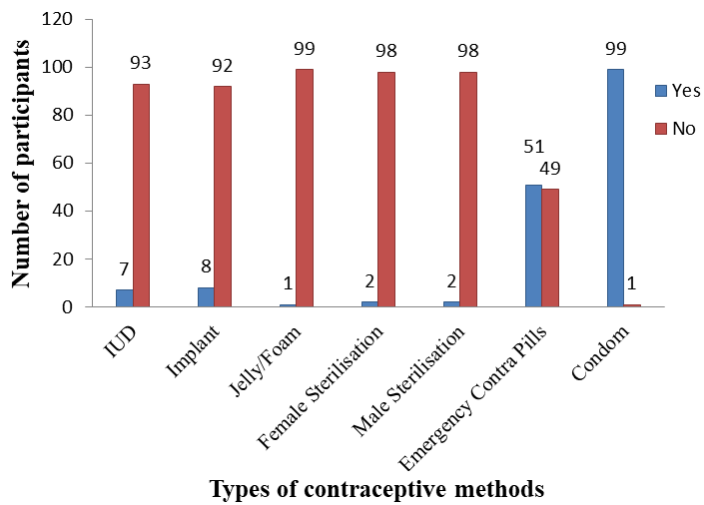
awareness on the various contraceptive methods among respondents

**Figure 2:**
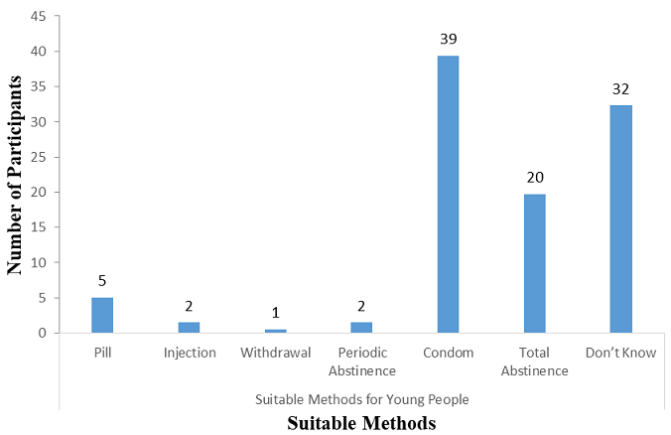
suitable methods of contraceptive method preferred by respondents

**Figure 3:**
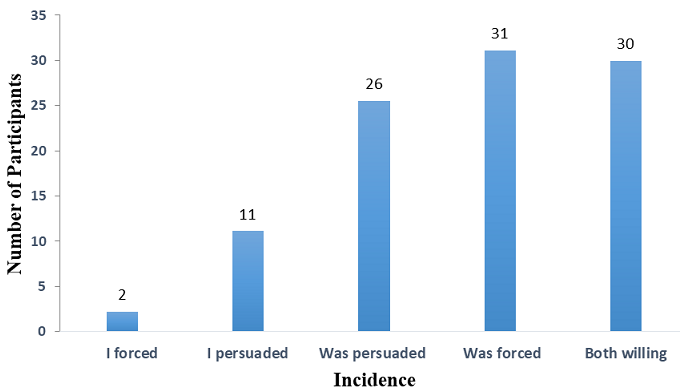
incidence regarding first time of sex

**Access to contraceptive methods among the AWD in the special needs schools:** in all 70.7% indicated they had heard of contraceptive methods. Figure 1 gives details on the various contraceptive methods and the awareness level among respondents who have ever heard about contraceptive. Only emergency pills and condoms were highly known among most of the respondents (51% and 99% respectively). There was low awareness on most of the contraceptive methods. Only emergency pills and condoms were highly known among most of the respondents (51% and 99% respectively). Condoms remained the most preferred contraceptive method for the respondents (39%) (Figure 2). Total abstinence was also preferred by 20% of the respondents. The obvious known barriers agreed by most of the respondents (45.0%) was embarrassment or feeling of shyness accessing the particular contraceptive (Figure 3).

**The sexual behaviors of Adolescents with disabilities:** in all, 54.6% of the respondents had ever been in a sexual relationship. It was indicated that having sex was higher among those who had been in any relationships before (86.7%) than those who had not (13.3%) and this was statistically significant (p-value<0.001; OR:4.69-21.16). The average age of those who had had sex was 13 years (+3.54). It was found that 76.7% of the sexually active respondents used no protection; only 23.3% confirmed using protection. Among those who used no protection, 86.4% confirmed not being concerned about HIV/AIDS whiles 13.6% were somewhat concerned. The majority (31%) of the respondents was forced into sex whereas or 30% were willing to engage in sex (Figure 3). Those who were persuaded or persuaded others formed 26% and 11% respectively. 76.7% of the sexually active respondents used no protection. Among those who used no protection, 86.4% confirmed not being concerned about HIV/AIDS (Table 5).

**Table 5:** barriers to contraceptive access among respondents in the four special schools

Variables	Yesn (%)	Non (%)
Not knowing where to go	32 (11.4)	248 (88.6)
Not knowing how to get there	26 (9.3)	254 (90.7)
Costly/Not able to pay for services	45 (16.1)	235 (83.9)
Privacy not respected	8 (2.9)	272 (97.1)
Not treated nicely by providers	4 (1.4)	276 (98.6)
Not being allowed to go alone afraid/Fearful	50 (17.9)	230 (82.1)
No same sex provider	1 (0.4)	279 (99.6)
Embarrassed/Feel shy	126 (45.0)	154 (55.0)
Inconvenient Hours/Days	6 (2.1)	274 (97.9)

Data is presented in frequencies and percentages Sources: Field data, 2018

## Discussion

The average age of respondents was 17 years with majority within the age group of 15 to 17 years of age. This corresponds with the standard age group of adolescents and various studies among adolescents have reported similar age ranges [4, 13, 14]. With regards to their various types of impairment, those with hearing disabilities were the majority (52.1%) followed by the visually impaired. In a similar studies conducted in parts of Nigeria among the same population group, it was found that the hearing impairment were also high [15, 16]. These findings were inconsistent with what was reported in the United States of America and in Europe where intellectual disabilities were found to be high [17, 18]. It was also found that majority of the special needs students could read and write (80%) and this findings were similar to Mbeba *et al*. [19] in a study done in Tanzania. In Ghana, reading and writing among disabled people have now become common, especially the visually impaired, since the introduction of assistive devices such as the Braille among others and this was reflective in the findings of this study. Also, with the introduction of Special Schools, most disabled children are receiving tuition and practices tailored to their particular needs.

**Sources of information on adolescent reproductive health:** sexual reproductive health information is essential to guide the adolescent to become aware of his or her sexuality in order to make the right decisions and avoid engaging in risky sexual behaviours. This current study revealed that school teachers were the major sources of information for the adolescent with disabilities (63.9%) followed by parents (12.2%). In contrast to the findings on sources of information found by this current study, Ngilangwa *et al*. [20] reported that the major sources of SRH information received by the adolescents were from their peers (36.7%) and the radio (22.5%).

**Knowledge of adolescents with disabilities on SRH:** the study discovered that 67.1% of the respondents had good knowledge regarding sexual and reproductive health which was consistent with a similar study in Tanzania [20]. It was again indicated that some demographic factors such as the age of the adolescent and religion were significantly associated with knowledge level (p-values= 0.026 and 0.034) respectively. Type of disability was also found to be fairly associated with knowledge level (p-value=0.057). Thus, those within 18-19 years were almost twice as likely to have a higher knowledge compared to those within 15-17 years (AOR:1.88; 95% CI:1.07-3.30). These findings indicated that age of the adolescent had a strong association with their knowledge level. Also, participants with visual impairments were 2.3 times more likely to have good knowledge compared with those with intellectual disability (AOR:2.37, 95%CI:1.03-5.49), and this was similar to what was reported by Kennedy *et al*, [15].

**Access and awareness of contraceptive methods:** when adolescents' awareness on contraceptives was studied, it was discovered that the majority of them (70.7%) were aware of at least one contraceptive method. Among all the contraceptives, awareness was high on condoms. Almost all of the special needs adolescents (99%) had heard or knew about condoms. In a study done in four sub-Sahara countries, it was also found that the male condom was the most widely known contraceptive by adolescents [21]. Those that were less known among them were IUD (7%), implant (8%), Jelly foams (1%) and male and female sterilisation (2% each). This was inconsistent with findings by Adejumo and Umoren [17] in Nigeria where IUD and Implant were also widely known among the adolescent.

**Sexual behaviors of AWD:** sexual behaviour of the adolescents are the various sexual practices and engagements by the adolescents. This study found that 54.6% of the adolescents had been in a relationship before and among these, females were the majority (51%). In Ghana and Africa at large, statistics show that sexual or opposite sex relationship begin early in girls than in boys [20]. In the same study, the majority of those who had been in a relationship before (90.2%) had engaged in some form of physical contacts such as kissing and touching. Similar findings were made by Tanabe *et al*. [22] in a study conducted among adolescent refugees on sexual risky behaviour assessment in Kenya, Nepal and Uganda. In that study, sexual intercourse was found to be among 32.1% of the adolescents with disability and common among 86.7% of those who had been in a relationship before; thus maintaining a relationship with the opposite sex was a predictor of having sex (p<0.001; OR:4.6; 95% CI:9-21.16).

## Conclusion

Most of the special needs adolescents in Ghana spend their time in schools, thus reflecting in their sources of information on adolescent sexual reproductive health. Knowledge level was found to be high, with an association on factors such as age and religion. Other factors such as sources of information and the type of guardian were also associated with knowledge level. Condoms were the obvious and the most known type of contraceptive among the studied adolescents. There was a low awareness of the other contraceptives such as IUD, implant, jelly among others. Increasing the awareness of the other contraceptive methods could help AWD to make the right choices in future. Barriers such as fear and the feeling of shyness was also found to influence their access to contraceptive methods. Sexual relationships were common among the studied participants; hence, a change in societal mindsets about adolescent sexuality, particularly those living with disability would be beneficial. Again, engaging in heterosexual relationships was a key factor to practicing sex. It is therefore imperative to give these groups of adolescents the right information and to make available friendly SRH services to meet for their SRH needs. It is recommended that steps and approach should be taken to integrate SRH education should into school curriculum of AWD, thereby enabling the gradual acquisition of information and knowledge necessary to develop the appropriate skills and favorable attitudes needed for a healthy reproductive life. It would also be essential to encourage guardians to show interest by engaging their adolescents disabled wards in SRH topics.

### What is known about this topic

- A study conducted in Ghana among visually impaired women indicated that teenagers often engage in pre-marital sex with no adequate knowledge to prevent unplanned pregnancies;
- Reports indicate that vulnerable groups among people with disabilities are women, children, elderly, refugees, displaced persons and migrant workers;
- Discriminations that women with disabilities often experience increase their vulnerability to different forms of violence.

### What this study adds

- School teachers are the major sources of information for the adolescent with disabilities in Ghana;
- Respondents had good knowledge regarding sexual and reproductive health;
- Although awareness of condoms was high, there were low levels of knowledge among IUD, implant, Jelly foams, as well as the male and female sterilisation.

## Competing interests

The authors declare no competing interests.
